# Exploring the potential of moringa leaf extract as bio stimulant for improving yield and quality of black cumin oil

**DOI:** 10.1038/s41598-021-03617-w

**Published:** 2021-12-20

**Authors:** Abid Mehmood, Khalid Naveed, Qasim Ayub, Saud Alamri, Manzer H. Siddiqui, Chao Wu, Depeng Wang, Shah Saud, Jan Banout, Subhan Danish, Rahul Datta, Hafiz Mohkum Hammad, Wajid Nasim, Muhammad Mubeen, Farooq Shah, Shah Fahad

**Affiliations:** 1 Department of Agronomy, The University of Haripur, Haripur, 22620 Khyber Pakhtunkhwa Pakistan; 2 Department of Horticulture, The University of Haripur, Haripur, 22620 Khyber Pakhtunkhwa Pakistan; 3 Department of Botany and Microbiology, College of Science, King Saud University, Riyadh, 11451 Saudi Arabia; 4 Guangxi Key Laboratory of Functional Phytochemicals Research and Utilization, Guangxi Institute of Botany, Guangxi Zhuang Autonomous Region and Chinese Academy of Sciences, Guilin, 541006 China; 5 College of Life Science, Linyi University, Linyi, 276000 Shandong China; 6 Faculty of Tropical AgriSciences, Czech University of Life Sciences Prague, Prague, Czech Republic; 7 Department of Soil Science, Faculty of Agricultural Sciences and Technology, Bahauddin Zakariya University, Multan, 60800 Pakistan; 8 Department of Geology and Pedology, Faculty of Forestry and Wood Technology, Mendel University in Brno, 61300 Brno, Czech Republic; 9 Department of Agronomy, Muhammad Nawaz Sharif University of Agriculture, Multan, 66000 Pakistan; 10 Department of Agronomy, University College of Agriculture and Environmental Sciences, IUB, Bahawalpur, Pakistan; 11 Department of Environmental Sciences, COMSATS University Islamabad, Vehari Campus, Islamabad, Pakistan; 12 Department of Agriculture, Abdul Wali Khan University Mardan, Khyber Pakhtunkhwa, 23200 Pakistan; 13 Hainan Key Laboratory for Sustainable Utilization of Tropical Bioresource, College of Tropical Crops, Hainan University, Haikou, 570228 Hainan China

**Keywords:** Ecology, Plant sciences

## Abstract

The history of plants to be utilized as medicines is thousands of years old. Black cumin is one of the most widely examined plant possessing naturally occurring compounds with antimicrobial potential. Foliar application of growth stimulators is a successful strategy to enhance yield and quality in many crops. A field study was planned to apply growth stimulator like moringa leaf extract on black cumin crop grown under field conditions using RCB design with three replications. All other agronomic inputs and practices were uniform. The treatments were moringa leaf extract concentrations (10%, 20%), growth stages (40 days after sowing, 80 DAS, 120 DAS, 40 + 80 DAS, 40 + 120 DAS, 80 + 120 DAS, 40 + 80 + 120 days after sowing) and two controls unsprayed check (i.e. no moringa leaf extract, no water) and sprayed check (no moringa leaf extract + water). Application of 20% moringa leaf extract at stage-7 (40 + 80 + 120 days after sowing) had significantly increased plant height, branches plant^−1^, essential oil content, fixed oil content, peroxidase value and iodine value of black cumin oil over unsprayed control. Application of moringa leaf extract showed maximum results and improves growth and yield of black cumin when applied at 40 + 80 + 120 days after sowing. As this study was only conducted using moringa leaf extract, it is advisable to conduct an experiment with various bio stimulants along with fertilizer combinations and growth regulators to check their synergistic effects for more reliable and acceptable recommendations in future.

## Introduction

Black cumin (*Nigella sativa* L.) also known as Kalonji locally, is one of the most widely examined plant possessing naturally occurring compounds with anti-cancer potential. Black cumin is a filed crop of family *Ranunculaceae* also known as butter cup family^1^. Its seeds are similar in appearance to onion seeds and used for medicine or for flavoring foods^2^. It is originated from Mediterranean region and spread to Asia, Africa and Europe^3^. The numerous vernacular names indicate the use of the spice in more than 100 countries. Nigella seeds are being utilized as a medicine and spice since the prehistoric time. Seeds have bitter and unpleasant taste so that the consumption of whole seed even in small quantity gives a feeling of constriction of throat^4^.

In Islam, black cumin is considered as the best healing medicine available. The Holy Prophet Muhammad (
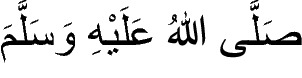
) once stated that “Kalonji can cure every disease except death”. Due to its higher nutritional values and medicinal importance it is also refereed as *Seed of blessing* (*habbatul barakah*)^5^. Medicinal value of black cumin is immense and numerous workers appreciated its unique, varied and powerful pharmacological traits. The medicinal importance of the herb is widely reported^6^. Oil of black cumin seeds is high in unsaturated fatty acids and can be predicted to have significant health benefits when consumed^7^. The most potent and fruitful medicinal properties lie in its volatile components^8^.

The production of black cumin in Pakistan has not been reported on commercial scale but it is widely used for various purposes in the country. A range of endogenous and external factors influence crop productivity and development. In addition, crop development is often influenced by a variety of biotic and abiotic stresses^9,10^.

Moringa is among one of its 13 species belongs to genus Moringa and family *Moringnance*. Moringa oils and leaves contains some most potent antioxidants and have higher concentrations of minerals like iron, calcium, phenolic acid and vitamins like vitamin A, C riboflavin and beta carotene^11^. The availability of mineral nutrients in moringa leaf might be used to complement the nutritional needs of horticulture crops, reducing the quantity of chemical fertilizer used. At very low or diluted concentrations, moringa leaf extract acts as a bio stimulant^12^. Moringa leaf extracts (MLE) are also have enhanced amounts of zeatin, which plants greener and imparts stress tolerance in plants^13^. Certain previous studies indicated the presence of many vital antioxidants in MLE but a very little information is available on activity of these antioxidants and mechanism which regulates the physiological process in black cumin crop.

## Results and discussion

### Plant height (cm)

Plant height of black cumin as affected by moringa leaf extract applied at various growth stages is reported in Table 1. Both concentrations of moringa leaf extract significantly affected plant height of black cumin. All growth stages also showed statistically significant results. Mean comparison of control vs treatments and water spray vs rest were also found significant for plant height (cm) of black cumin. Whereas, interaction of moringa leaf extract concentrations and growth stages remained non-significant. With increase in interval of spraying moringa leaf extract, plant height enhanced and thus taller plants (68.15 cm) were recorded when moringa leaf extract was sprayed at stage-7 (40 + 80 + 120 days after sowing), followed by (65.15 cm) stage-4 (40 + 80 days after sowing), while lower plants height (47.45 cm) was recorded in stage-3 (120 days after sowing). The use of moringa leaf extract during critical vegetative development phases increased the black cumin crop's plant height. Similar results were recorded by Abbas et al.^14^ that moringa leaf extract enhanced plant height and improved fresh and dried weight of wheat root when compared to control. Taller (62.2 cm) plants were recorded in 20% moringa leaf extract sprayed plots followed by (55.8 cm) 10% moringa leaf extract. Spraying moringa leaf extract on a variety of field crops can boost plants and increase vegetative development^15^.

**Table 1:** Plant height (cm), number of branches plant^−1^ fixed oil content (% vw^−1^) and essential oil content (% vw^−1^) of black cumin as affected by moringa leaf extract applied at various growth stages.

	Plant height (cm)	Number of branches plant^−1^	Fixed oil content (% vw^−1^)	Essential oil content (% vw^−1^)
**Growth stages**				
Stage 1	51.8	55.16	32.68	0.36
Stage 2	61.45	62.5	34.37	0.35
Stage 3	47.45	51.66	33.41	0.37
Stage 4	65.15	65.5	35.18	0.39
Stage 5	54.9	62	34.64	0.36
Stage 6	64.13	60.83	35.72	0.37
Stage 7	68.15	70.66	37.08	0.42
LSD (0.05)	1.15	1.42	0.33	0.007
**MLE concentrations**				
MLE @ 10%	58.83	60.19	34.06	0.37
MLE @ 20%	62.2	62.19	35.39	0.38
LSD (0.05)	0.32	1.42	0.09	0.002
**Control vs rest treatments**				
Control	42.7	39	31.48	0.33
Rest treatments	58.02	61.19	34.73	0.37
Significance	**	**	**	**
**Water only vs rest treatments**				
Water only	44.2	40.33	33.29	0.34
Rest treatments	58.02	61.19	34.73	0.37
Significance	**	**	**	**
**Interaction**	**Significance**			
Stages × concentrations	NS	Figure 1	Figure 2	NS

*MLE* moringa leaf extract, *@* at the rate, *%* percent, *Stage 1 *application of MLE at 40 DAS, S2 = Application at 80 DAS, S3 = 120 DAS, S4 = 40 + 80 DAS, S5 = 40 + 120 DAS, S6 = 80 + 120 DAS, S7 = 40 + 80 + 120 days after sowing, **highly significant, *NS* non-significant, *LSD* least significant difference.

### Branches plant^−1^

**Figure 1:**
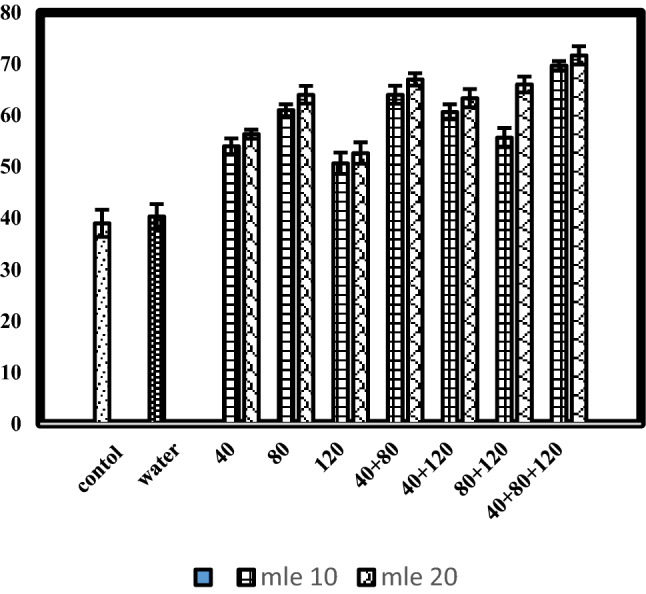
Number of branches plant^−1^ of black cumin as affected by moringa leaf extract applied at various growth stages.

Branches plant^−1^ of black cumin were significantly influenced by moringa leaf extract concentrations, stage of application as well as their interaction (Table 1). The planned mean comparison of control vs rest and water spray vs rest were also found significant for branches plant^−1^. The unsprayed against sprayed treatments of moringa leaf extract showed that in unsprayed plots number of branches plant^−1^ (39) were less than plants sprayed with moringa leaf extract (61.19). Highest number of branches plant^−1^ (62.19) were observed 20% moringa leaf extract treated plots. These results are in agreement with Mahmood^16^ who found that foliar application of MLE contains an adequate amount of stimulating substances that promote cell division and enlargement at a faster rate. Zeatin, a growth hormone found in moringa leaf extract, encourages the growth of lateral buds, which leads to an increase in the number of branches. After pounding 100 g of Moringa leaves in 8 L of water, foliar spray of moringa leaf extract enhanced branches plant^−1^ in okra^17^. More number of branches plant^−1^ (70.66) were attained in plots sprayed with moringa leaf extract at growth stage 7 (40 + 80 + 120 days after sowing), followed by growth stage 4 (40 + 80 days after sowing). The effect of the application of MLE at the rate of 20% at 40 days’ interval increased the number of branches and this may be because of the abundant supply of macro and micronutrients and growth hormones. The result of yield parameters revealed that the yield increased as the frequency of moringa leaf extract increased. This is because hormone enhances formation and development of flowers and ripening of fruits. Hormones also enhance growth and yield by altering photosynthetic distributive pattern within the plants. The findings were also in line with that of Manzoor et al*.*^18^ who found that an aqueous extract of moringa significantly influence yield and yield components such as number of branches, number of fruits per plant and fruit weight of tomato. The significant interaction of MLE and growth stages is presented in Fig. 1. Applying moringa leaf extract @ 20% at all growth stages enhanced branches plant^−1^. Maximum branches plant^−1^ was observed when moringa leaf extract was sprayed @ 20% at growth stage 7 (40 + 80 + 120 days after sowing) whereas, minimum branches plant^−1^ was recorded in plants sprayed with 10% moringa leaf extract at growth stage-3 (120 days after sowing). Moringa leaf extract (MLE) increased number of branches. Similar results were recorded by Jain et al.^19^), who reported MLE positively enhanced plant growth attributes of wheat. He also stated that with increasing MLE concentration and application intervals, the growth parameters such as branches plant^−1^ were increased in arithmetic order. Plant growth regulators are essential for controlling growth and development of plants^20^. These plant growth regulators increased yield by changing the dry matter distribution pattern or controlling the growth characteristics in crop plants, depending on the dosage and time of application^21^. In comparison to control, foliar application of moringa leaf extract resulted in a markedly higher branches plant^−1^. The increased number of branches plant^−1^ might be due to Zeatin present in moringa leaf extract, which is very effective in delaying the abscission response^10^.

### Fixed oil content (% vw^−1^)

**Figure 2:**
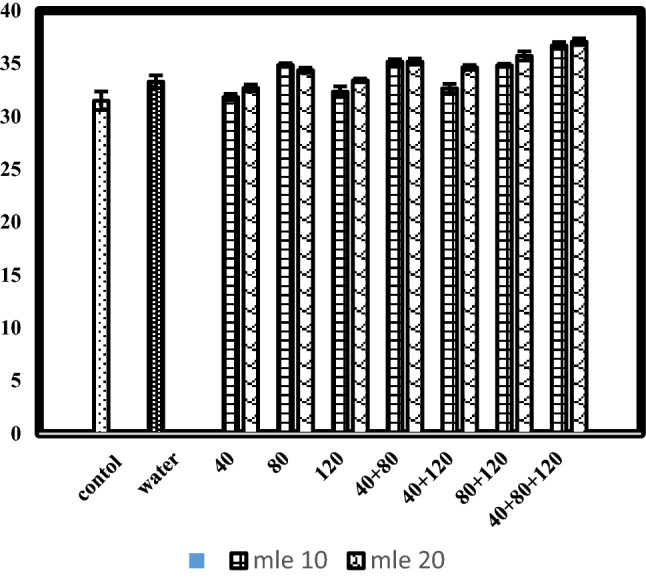
Fixed oil content (%) of black cumin as affected by moringa leaf extract applied at various growth stages.

Data concerning fixed oil content (% vw^−1^) in response to moringa leaf extract applied at various growth stages is given in Table 1 and Fig. 2. Statistical analysis of data indicated that foliar application of various concentrations of moringa leaf extract, their stage of application and interaction of concentrations and growth stages had significantly affected fixed oil content (% vw^−1^) of black cumin crop. The planned mean comparison of control vs rest and water spray vs rest had significant effect on fixed oil content (% vw^−1^). Highest fixed oil percentage (35.39%) was recorded when moringa leaf extract was sprayed @ 20%, followed by (34.06%) 10% moringa leaf extract, whereas, control (31.48%) showed lowest fixed oil %. Sakr et al.^22^ indicated that foliar applications of MLE significantly improved the oil percentage and yield plant^−1^ and feddan of geranium plants. Application of MLE at growth stage-7 (40 + 80 + 120 days after sowing) showed maximum fixed oil content percentage (37.08%) as compared to all other growth stages. Minimum fixed oil percentage was recorded in growth stage-1 (40 days after sowing). Concerning the interaction of moringa leaf extract vs application stage, highest fix oil (37.45%) was observed when moringa leaf extract @ 20% was applied as foliar spray at growth stage-7 (40 + 80 + 120 days after sowing), followed by (36.71%) moringa leaf extract @ 10% applied at growth stage-7. Lowest fixed oil percentage (31.83%) was observed in plants sprayed with 10% moringa leaf extract at stage 1 (40 days after sowing). According to Rady et al.^23^, biosynthesis of cytokinins promotes the movement of stem reserves to new shoots, resulting in stable plant development, the prevention of premature leaf senescence, and the preservation of more leaf area for photosynthetic action.

### Essential oil content (% vw^−1^)

Essential oil content (% vw^−1^) is a vital oil component of black cumin. Moringa leaf extract concentrations and stage of their application had significant effect on essential oil content of black cumin while the interaction remained non-significant (Table 1). Application of MLE at 20% resulted in higher essential oil yield (0.38%) followed by 10% moringa leaf extract (0.37) sprayed plots. Control plots resulted in lower essential oil (0.33%) content of black cumin. Many research ventures around the world are currently focusing on increasing the biomass yield and volatile oil output of aromatic plants. Moringa leaf extract has been discovered to be an excellent bio-stimulant for enhancing not only crop growth but also yield^24,25^. According to Aslam et al.^26^, Plant treated with MLE had major impacts, including an average rise in oil concentrations. Interestingly, MLE treatment not only increased the coriander fruit yield but also improved the fruits volatile oil suggesting that MLE could be a promise plant growth promoter that improved the content of volatile oil in coriander. MLE application also positively affected the volatile oil constituents (Table 2). Increasing the volatile oil in coriander by MLE could be due to the MLE components including amino acids, nutrient elements and phytohoromes that motivate the accumulation of secondary metabolites^27^. The phytohormones affect the pathway of terpenoids through motivating the responsible physiological and biochemical processes^28^. Concerning the application stages of moringa leaf extract, higher essential oil content % of black cumin (0.42%) was observed in growth stage-7 (40 + 80 + 120 days after sowing), followed by (0.39%) growth stage-4 (40 + 80 days after sowing), whereas, lower essential oil content % (0.36%) of black cumin was observed in growth stage-1 (40 days after sowing). Plant growth regulators are essential for controlling the amount, type, and direction of plant growth, development, and yield^20^. These plant growth regulators increased yield by changing the dry matter distribution pattern or controlling the growth characteristics in crop plants, depending on the dosage and time of application^21^. Exogenous application of MLE resulted in higher yield and quality^29^.

**Table 2:** Peroxidase value (meq kg^−1^) and Iodine value (g of I_2_/100 g) of black cumin as affected by moringa leaf extract applied at various growth stages.

	Peroxidase value (meq kg^−1^)	Iodine value (g of I_2_/100 g)	Total phenolic	Total free amino acids
**Growth stages**				
Stage 1	6.3	82.83	69.25	314.16
Stage 2	6.02	84.52	73.85	323.23
Stage 3	6.12	84.57	55.25	290.31
Stage 4	5.73	86.95	63.46	355.93
Stage 5	6.22	83.67	71.38	323.23
Stage 6	6.39	85.61	76.66	345.45
Stage 7	6.42	87.35	81.23	364.28
LSD (0.05)	0.077	0.25	0.82	3.2
**MLE concentrations**				
MLE @ 10%	6.03	84.84	68.73	325.54
MLE @ 20%	6.30	85.3	71.59	336.34
LSD (0.05)	0.011	0.072	0.23	0.91
**Control vs rest treatments**				
Control	5.43	78.28	50.66	265.96
Rest treatments	6.171	85.0747619	69.19	327.16
Significance	**	**	**	**
**Water only vs rest treatments**				
Water only	5.23	80.91666667	55.73	274.16
Rest treatments	6.171	85.0747619	70.15	330.94
Significance	**	**	**	**
**Interaction**	**Significance**			
Stages × concentrations	Figure 3	Figure 4	NS	NS

*MLE* moringa leaf extract, *@* at the rate, *%* percent, *Stage 1* application of MLE at 40 DAS, S2 = Application at 80 DAS, S3 = 120 DAS, S4 = 40 + 80 DAS, S5 = 40 + 120 DAS, S6 = 80 + 120 DAS, S7 = 40 + 80 + 120 Days after sowing, **highly significant, *NS* non-significant, *LSD* least significant difference.

### Peroxidase value (meq kg^−1^)

**Figure 3:**
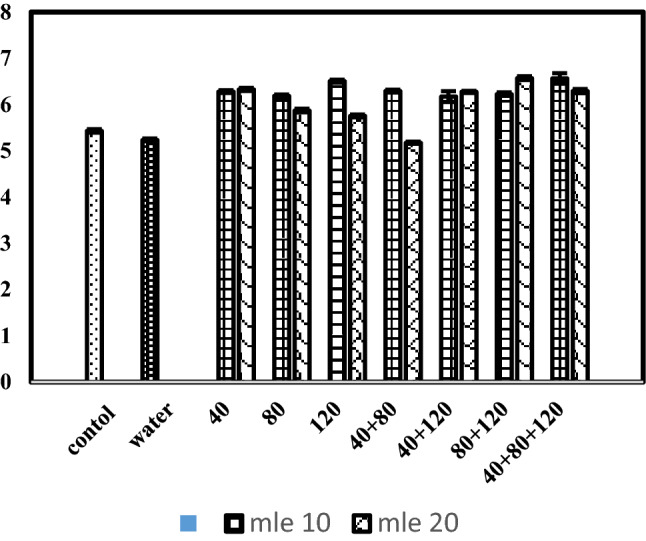
Peroxidase value (meq kg^−1^) of black cumin as affected by moringa leaf extract applied at various growth stages.

The response of MLE and stage of MLE application recorded for peroxidase value is stated in Table 2. The data depicted that moringa leaf extract concentrations, stage of application and their interaction had significant (P ≤ 0.05) variation in peroxidase value of black cumin. Similarly, when means were compared, that of control vs treatments and water spray check vs treatments were found significant for peroxidase value (%). Mean value of data indicated that highest peroxidase value (6.32%) was recorded in 20% moringa leaf extract treated plots, followed by (6.03%) 10% moringa leaf extract. While in case of application stages, highest peroxidase value (6.42%) was recorded when moringa leaf extract was applied at stage-7 (40 + 80 + 120 days after sowing), followed by (6.39%) stage-6 (80 + 120 days after sowing). Whereas lowest peroxidase value (5.73%) was recorded in plots treated with moringa leaf extract at stage-3 (120 days after sowing). Interaction of moringa leaf extract concentrations and stage of application in Fig. 3 showed that increasing moringa leaf extract concentration from 10 to 20% applied at growth stage-7 increased peroxidase value of black cumin crop. However, application of moringa leaf extract @ 10% applied at growth stage-3 (120 days after sowing) showed lowest peroxidase value. The phytohormones affect the pathway of terpenoids through motivating the responsible physiological and biochemical processes^28^. Our results are in agreement with the reports of Ali et al.^27^ in geranium and Abdel-Rahman and Abdel-Kader^30^ in fennel who observed that MLE application improves both the volatile oil yield and its components. The fact that MLE application improved black cumin growth and quality characters suorts the study's hypothesis that MLE is an important plant growth enhancer. In agreement with our results, Rady and Mohamed^28^ concluded that MLE is considered one of the important plant bio stimulants because it contains antioxidants, phenols, basic nutrients, ascorbates, and phytohormones. Furthermore, foliar application of moringa leaf extract may have a positive effect on endogenous phytohormone concentrations, resulting in improved plant growth and quality^10,37^.

### Iodine value (g of I_2_/100 g)

**Figure 4:**
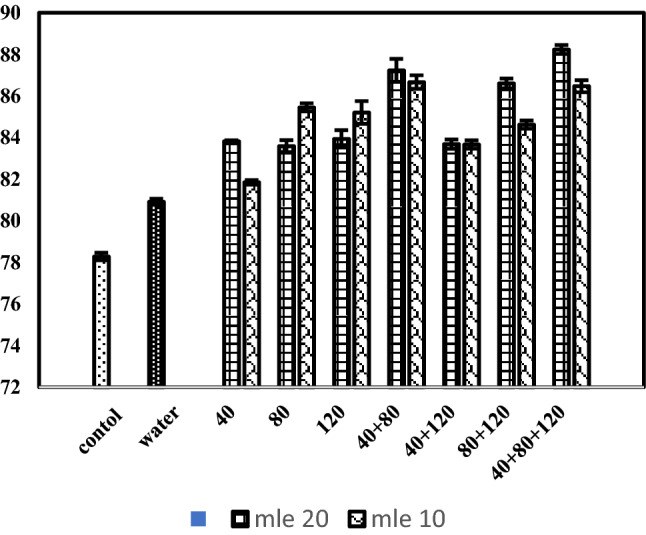
Iodine value (meq kg^−1^) of black cumin as affected by moringa leaf extract applied at various growth stages.

Data concerning iodine value of black cumin oil in response to various concentrations of MLE applied at various growth stages is given in Table 2 and Fig. 4. Statistical analysis of data indicated that both the concentrations of moringa leaf extract, stage of application as well as their interaction had significant effect on iodine value of black cumin oil. The planned mean comparison of control vs rest and water spray vs rest treatments had significant effect on iodine value. Highest iodine value (85.3) was recorded with application of moringa leaf extract @ 20% whereas, lowest (78.28) was observed in control. Regarding the stage of application, highest iodine value (87.35) was observed in plots sprayed with moringa leaf extract at stage-7 (40 + 80 + 120 days after sowing), followed by (85.61) plots sprayed with moringa leaf extract at growth stage-6 (80 + 120 days after sowing). Concerning the interaction of MLE concentrations and stage of application of MLE, highest iodine value (6.49) was observed with 20% moringa leaf extract sprayed at stage-7 (40 + 80 + 120 days after sowing) whereas, lowest iodine value was observed in plants sprayed with moringa leaf extract @ 20% applied at stage-3 (120 days after sowing). The use of plant growth regulators is very specific and depends to achieve specific results like for example; enhanced plant growth, betterment in yield and yield related attributes, and to modify the fruit and plant bio-constituents. Several previous studies reveled that MLE are enriched with many phtyo-hormones especially zeatin^31^. In addition to that MLEs are embedded with many essential amino acids, vitamins (A, B_1_, B_2_, B_3_, C and E), minerals as well as several antioxidants like phenolic^32,33^. This unique biochemical composition of MLE showed that they can be utilized as bio stimulant which have the potential to promote crop growth, productivity as well as quality which in return depends on its application time^34^.

### Total free amino acids

The data presented in Table 2 revealed that moringa leaf extract concentrations and application stages had significantly affected total free amino acid content of black cumin crop during rabi 2019-20 under agro-climatic conditions of Haripur whereas, their interaction remained non-significant. The planned mean comparison of control vs rest and water spray vs rest had significant effect on total free amino acids of black cumin. Highest amino acids (336.3) were observed with the application of moringa leaf extract @ 20%, followed by application of moringa leaf extract @ 10%. Regarding application stages, highest total free amino acids (364.2) were observed with the application of moringa leaf extract at 40 + 80 + 120 days after sowing, followed by (355.9) application of MLE at 40 + 80 days after sowing. Lowest total free amino acids (290.3) were recorded with moringa leaf extract sprayed at 40 days after sowing. Several investigations have demonstrated that MLE can alter both primary and secondary metabolism, resulting in an increase in antioxidant molecule concentrations^35,36^. The content of phenolic antioxidants, total soluble proteins, and total free amino acids increased in spinach plants treated with synthetic growth regulators and MLE^26^. MLE can also increase fruit quality metrics in ‘Kinnow' mandarins, such as soluble solid contents, vitamin C, sugars, total antioxidant, phenolic contents, and superoxide dismutase and catalase enzyme activities, when treated at various growth stages^37^.

### Total phenolic

Phenolic have acquired much importance because of their properties of disease preventing and health promoting. The effect of moringa leaf extract concentrations, stage of application and their interaction is presented in Table 2. Analysis of variance revealed that moringa leaf extract concentrations and stage of application of moringa leaf extract had significant effect on total phenolic content of black cumin while their interaction remained non-significant. Our results depict that all MLE levels enhanced the total phenolic content of black cumin leaves relative to the control. Highest phenolic content (71.59 mg g^−1^) was observed with application of moringa leaf extract at the rate of 20%, followed by (68.72 mg g^−1^) moringa leaf extract application at the rate of 10%. Regarding application stages, highest phenolic content (81.23 mg g^−1^) was observed with the application of moringa leaf extract at growth stage-7 (40 + 80 + 120 days after sowing), followed by (76.66 mg g^−1^) stage-6 (80 + 120 days after sowing), whereas, lowest phenolic content (55.25 mg g^−1^) was observed in crop sprayed with moringa leaf extract at stage-3 (120 days after sowing). In the medicinal, biological, and agricultural areas, phenolic and their derivatives gained scientists attention. Recent studies had focused on their potential as antioxidant-rich natural chemicals^38^. The increased content of phenolics, flavonoids, and phytohormones in moringa leaves, which may have contributed to the enhanced total phenolic content in black cumin leaves, can be linked to the higher content of phenolics, flavonoids, and phytohormones in MLE treated plants^26^. Furthermore, the proper concentrations of minerals, vitamins, and -carotene found in moringa leaves may have influenced metabolic processes in a way that increased the internal phenolic content in black cumin leaves, either directly or indirectly^39^. Therefore, these aspects assist MLE to serve as growth enhancer and natural antioxidant^40^. Our results supported by the previous report of Nasir et al.^37^ who revealed that the total phenolic content was enhanced as a result of MLE application at critical stages of plant growth.

## Materials and methods

A field experiment was carried out during 2019–2020. The treatments were arranged in RCB design with factorial arrangements and replicated three times. Size of plots were kept 2.7 × 1.5 m^2^. Uniform and normal agronomic practices were kept for all the treatments throughout the study.

### Source of moringa leaves

Moring leaves and branches were harvested from fully grown and mature Moringa trees planted at Horticulture Nursery, The University of Haripur.

### Preparation of MLE

Stock solution of MLE was made by the method described by Price^41^. Plant material was grinded with the help of locally fabricated extraction machine, for extraction 1 L of water is added to 10 kg of plant material. After extraction whole mixture was sieved through a cheese cloth to purify the stock solution of MLE. This stock was further diluted with water (distilled) to make several concentrations for foliar spray.

### Observations on the crop

#### Plant height (cm)

Plant height at first flowering was measured when the selected plants for data collection from each plot at maturity.

#### Number of branches plant^−1^

The total number of branches from ten randomly chosen plants was counted at harvest time and average number of branches plant^−1^ was calculated.

#### Total free amino acids

Moore and Stein^42^ method was used for the determination of total free amino acids. In 10 mL of citrate buffer, fresh leaves (0.5 g) were homogenized (pH 5.0). The mixture was then centrifuged for 10 min at 15,000×*g*. The solution's optical density was measured at 570 nm using a spectrophotometer (Unico-UV 2100 Japan).

### Total phenolics

Julkunen-Tiitto^43^ method was used for the determination of total phenolic. With 2.5 mL of the Folin Ciocalteau reagent (10%), 1 mL of diluted extracts was oxidized, followed by neutralization with 2 mL of sodium carbonate (7.5%). The mixture was kept dark for 45 min and the absorption was measured using a spectrophotometer (Unico-UV 2100 Japan) at a wavelength of 765 nm. Gallic acid was used as standard.

#### Essential oil content (%)

Essential oil content of was determined by following Stainier^15^ method. Oil from hundred grams of crushed Kalonji seeds were extracted using hydro-distillation in 0.5 L distilled water for 3 h.

#### Fixed oil ratio (%)

Four grams of powdered Kalonji seeds sample were extracted with *n*-hexane for 6 h using soxlet aaratus for determining the fixed oil content (%).

#### Peroxide value (meq kg^−1^)

The peroxide values were calculated by the method of Jacob^16^ 0.5 g of black cumin oil was added to mixture of glacial acetic acid and chloroform (60% + 40% respectively). This solution was reacted with potassium iodide solution (0.5 mL) in a glass store flask. Later on this solution was mixed for 2 min on a shaker, after shaking 30 mL of distilled water was added and iodine obtained was treated with 0.01 N sodium thiosulfate by using starch solution as indicator. Then following formula was used to calculate peroxide and expressed as mille moles per kg oil.

$$\mathrm{Peroxide \,value }=\frac{0.5 \times \mathrm{ N }\times \mathrm{V }\times 100}{\mathrm{Weight \,of \,sample}}$$

where N = Normality of sodium thiosulfate solution V = Volume in mL of sodium thiosulfate needed for titration.

#### Iodine value (g of I_2_/100 g)

The unsaturation of black cumin oil was obtained by calculating the total amount of halogen absorption by oil as mentioned by Shantha and Decker^44^. 0.1–0.5 g of oil was added to mixture of chloroform (10 mL) and Hanus iodine (25 mL) solution and left for 30 min. after that 15% potassium iodine solution (10 mL) and freshly boiled and cooled distilled water (100 mL) was added to the mixture. Liberated iodine was titrated with 0.1 N of sodium thiosulfate using starch indicator.

$$\mathrm{Iodine \,value }= \frac{(\mathrm{V}1-\mathrm{V}2)\times \mathrm{N}\times 12.69}{\mathrm{Weight \,of \,sample}}$$

where N = Normality of sodium thiosulfate solution V1 = Volume in mL of sodium thiosulfate needed for titration Blank V2 = Volume in mL of sod. Thiosulfate needed for titration sample.

### Statistical analysis

The treatments mean comparison were made using least significant differences test for significant parameters at 0.05% level of probability where p values are significant (Gomez and Gomez^45^).

### Complies with international, national and/or institutional guidelines

Experimental research and field studies on plants (either cultivated or wild), comply with relevant institutional, national, and international guidelines and legislation. Experimental studies were carried out in accordance with relevant institutional, national or international guidelines or regulation.

### Identification of the plant material

Before collection, the plant was identified by Dr. Hanif Khan (Taxonomist), using the standard protocol at the Department of Soil Science, Agricultural University, Peshawar, Pakistan.

### Ethics approval and consent to participate

We all declare that manuscripts reporting studies do not involve any human participants, human data, or human tissue. So, it is not applicable.
